# Identification of β-Dystrobrevin as a Direct Target of miR-143: Involvement in Early Stages of Neural Differentiation

**DOI:** 10.1371/journal.pone.0156325

**Published:** 2016-05-25

**Authors:** Maria Teresa Quaranta, Isabella Spinello, Rosa Paolillo, Gianfranco Macchia, Alessandra Boe, Marina Ceccarini, Catherine Labbaye, Pompeo Macioce

**Affiliations:** 1 Department of Hematology, Oncology and Molecular Medicine, Istituto Superiore di Sanità, Rome, Italy; 2 Department of Cell Biology and Neurosciences, Istituto Superiore di Sanità, Rome, Italy; 3 National Centre for Rare Diseases, Istituto Superiore di Sanità, Rome, Italy; Rutgers University -New Jersey Medical School, UNITED STATES

## Abstract

Duchenne Muscular Dystrophy, a genetic disorder that results in a gradual breakdown of muscle, is associated to mild to severe cognitive impairment in about one-third of dystrophic patients. The brain dysfunction is independent of the muscular pathology, occurs early, and is most likely due to defects in the assembly of the Dystrophin-associated Protein Complex (DPC) during embryogenesis. We have recently described the interaction of the DPC component β-dystrobrevin with members of complexes that regulate chromatin dynamics, and suggested that β-dystrobrevin may play a role in the initiation of neuronal differentiation. Since oxygen concentrations and miRNAs appear as well to be involved in the cellular processes related to neuronal development, we have studied how these factors act on β-dystrobrevin and investigated the possibility of their functional interplay using the NTera-2 cell line, a well-established model for studying neurogenesis. We followed the pattern of expression and regulation of β-dystrobrevin during the early stages of neuronal differentiation induced by exposure to retinoic acid (RA) under hypoxia as compared with normoxia, and found that β-dystrobrevin expression is regulated during RA-induced differentiation of NTera-2 cells. We also found that β-dystrobrevin pattern is delayed under hypoxic conditions, together with a delay in the differentiation and an increase in the proliferation rate of cells. We identified miRNA-143 as a direct regulator of β-dystrobrevin expression, demonstrated that β-dystrobrevin is expressed in the nucleus and showed that, in line with our previous in vitro results, β-dystrobrevin is a repressor of synapsin I in live cells. Altogether the newly identified regulatory pathway miR-143/β-dystrobrevin/synapsin I provides novel insights into the functions of β-dystrobrevin and opens up new perspectives for elucidating the molecular mechanisms underlying the neuronal involvement in muscular dystrophy.

## Introduction

The dystrophin-related and associated protein dystrobrevin is a cytoplasmic component of the dystrophin-associated protein complex (DPC), a membrane complex that serves both structural and signaling functions [1,2] in muscle and non-muscle tissues. In muscle the DPC consists of dystrophin, dystroglycan, sarcoglycans, α-dystrobrevin and syntrophin (for a review see [3]), and its disruption due to a failure to assemble correctly is central to the pathogenesis of muscular dystrophy. In brain, the presence of several dystrophin isoforms, alternatively spliced variants and other DPC components, such as β-dystrobrevin and γ-syntrophin, which are not expressed in muscle, accounts for the existence of complexes that are more accurately described as DPC-like [4]. By analogy to the situation in muscle, defects in a neuronal DPC-like complex may contribute to the etiology of mild to severe cognitive impairment and learning disability that are a consistent finding in approximately one-third of patients with Duchenne (DMD) and Becker (BMD) muscular dystrophies [5,6]. The dystrobrevin protein family comprises the products of two different genes coding for two homologous proteins, α- and β-dystrobrevin [7,8]. α-Dystrobrevin is expressed predominantly in muscle and brain whereas β-dystrobrevin is expressed in non-muscle tissues such as brain, kidney, lung and liver [9]. Although their cellular functions are beginning to be elucidated through the study of their associated proteins, the exact role of dystrobrevins remains unclear. Mice lacking α-dystrobrevin exhibit a mild muscular dystrophy but no obvious central nervous system (CNS) defects [10], and mice lacking β-dystrobrevin are outwardly normal [11]; interestingly, double knock-out mice lacking both α- and β-dystrobrevin have a mild myopathy plus synaptic defects and abnormal motor behavior [12]. This provides evidence that DPC components other than dystrophin influence the synaptic structure in certain areas of the brain, and suggests that motor deficits in dystrophic patients may reveal not only peripheral but also CNS defects [12]. It has been reported that dystrobrevin forms DPC-like complexes in brain [13], where it associates with the dystrophin short isoform Dp71 in different subcellular compartments, including the nucleus [14]. Besides dystrophin and syntrophin, a number of specific partners of dystrobrevin have been described in the last two decades [15–19]. Among these, nuclear chromatin remodeling proteins iBRAF/HMG20a and BRAF35/HMG20b [20], which have been shown to play a central role in the regulation of neuronal differentiation [21,22]. These interactions suggest that in neurons dystrobrevin may have a role in the early processes of differentiation [20], opening a new scenario that would help in understanding the molecular mechanisms underlying the neuronal involvement in DMD.

Recently, oxygen concentrations also have emerged as crucial signaling factors related to neuronal differentiation [23]. The physiological concentration of oxygen in tissues is significantly lower than the 21% atmospheric oxygen, and tissue normoxia varies widely in human brain, as well as in other tissues. Hypoxia can therefore be considered not only a pathological event but also a physiological condition, modulating cellular processes like cell proliferation and differentiation, for instance [24]. The response to hypoxia involves the activation of hypoxia-inducible factors (HIFs), transcriptional factors responsible for the adaptation of cells to low oxygen tension, for the regulation of glucose metabolism and for cell proliferation and survival [25]. The activity of HIFs results in alterations in gene transcription of direct target genes and hypoxia-regulated microRNAs (HRMs) [26]. MicroRNAs (miRNAs) are small single stranded RNA molecules that bind to their target mRNA and post-transcriptionally regulate their target genes by inducing decay of target mRNA or suppressing translation. miRNAs are not only essential for neuronal differentiation and maintenance of neuronal phenotype [27–29] but also implicated in brain development, function and related disorders [30]. Investigations in several model organisms are beginning to show intricate regulatory networks involving miRNAs, transcription factors, and epigenetic regulators during CNS development [31]. It has been recently reported in Drosophila muscular dystrophy models that there is indeed a DPC-dependent circuitry of processes, evolutionarily conserved and present in neuronal tissue, which, through the control of miRNAs, is involved in the regulation of gene expression in dystrophic conditions [32]. The same group has also found that dystroglycan, a DPC component involved in the control of neuron behavior in addition to its function in muscle, is regulated by miRNAs during brain differentiation [33].

Uncovering the processes that underlie the role of oxygen concentration and/or specific miRNAs in regulating the expression of DPC components during the early stages of neuronal differentiation could shed new light on the importance of a correct DPC function in nervous system development and maturation. In this context, we have investigated the impact of oxygen concentration on the expression and regulation of β-dystrobrevin during short term retinoic acid (RA)-treatment of NTera-2 cl. D1 (NT2/D1) pluripotent human embryonal carcinoma cells, and observed that in hypoxia neural differentiation of these cells is impaired and β-dystrobrevin expression delayed, as compared with normoxia. Moreover, we identified β-dystrobrevin as a specific target of miR-143, and found evidence suggesting that the miR-143/β-dystrobrevin axis may play a role in neuronal proliferation and differentiation. Our results also demonstrated a role of β-dystrobrevin in the transcriptional control of synapsin I expression, in agreement with previous data obtained in vitro [20]. Synapsin I is a member of a family of neuron-specific phosphoproteins that regulates neurotransmission and proper neuronal development [34] and alterations in its expression have been associated to mental retardation, among other disorders [35,36]. Overall, our data strengthen the hypothesis of a functional involvement of DPC component β-dystrobrevin during the first steps of neural differentiation that might be relevant in the pathogenesis of the cognitive disability observed in DMD patients.

## Materials and Methods

### Cell Culture and Induction of Differentiation

Human embryonal carcinoma Ntera-2 cl. D1 (NT2/D1) cell line was purchased from ATCC (American Type Culture Collection, Manassas, VA) and maintained in Dulbecco’s modified Eagle’s medium (DMEM; Sigma-Aldrich, St Louis, MO, USA) supplemented with 10% heat inactivated FBS (Sigma), 4 mM glutamine, 100 units/ml penicillin, and 100 μg/ml streptomycin (Life Technologies, Carlsbad, CA) at 37°C in 5% CO_2_. Differentiation was started by seeding cells at 1x10^6^ cells per 75-cm^2^ flasks in 10^−5^ M trans-retinoic acid (RA; Sigma) from day 0 (which represents RA-untreated control cells) to day 12, as described [37–39]. Culture of untreated and RA-treated NT2/D1 cells was carried out under hypoxic and normoxic conditions. Human leukemic U937 cells were grown in RPMI medium supplemented with 10% FCS, under hypoxic conditions and used as control for hypoxia experiments. 293T cells grown in normoxia were used for luciferase assays.

### Hypoxia

To provide a hypoxic environment (1% O_2_), cells were cultured and treated in humidified 5% CO_2_ multigas incubators (Carbon Dioxide Incubator with oxygen and nitrogen control; New Brunswick Eppendorf, Hamburg, Germany), flushed continuously with a N_2_ gas to maintain established atmospheric O_2_ concentrations at a constant temperature of 37°C. Incubators were calibrated to 1% O_2_, 94% N_2_ and 5% CO_2_ referred to as hypoxia. For physiological oxygenation or normoxia, cells were cultured in an incubator calibrated to 5% CO_2_ in air humidified atmosphere (21% O_2_). Cell proliferation and viability of cells were evaluated by standard procedures such as cell counting and Trypan blue staining.

### RNA extraction and quantitative real-time PCR

Total RNAs were extracted using TRIzol reagent and reverse transcribed by Moloney murine leukemia virus reverse transcriptase (Invitrogen, Carlsbad, CA, USA) with random primers, as described previously [40]. HIF-1α, β-dystrobrevin (β-DB), synapsin I (SynI), NF-L and MAP2 mRNAs were detected by quantitative real-time PCR analysis (qRT-PCR) and normalized with the internal control β-actin (ACTB), using commercial ready-to-use primers/probe mixes for HIF-1α (Hs00153153_m1), β-dystrobrevin (DTNB) (Hs01027264_m1), SYNI (Hs00199577_m1), NF-L (Hs00196245_m1), MAP2 (Hs00258800_m1) and ACTB (20X, 4310881E) (Applied Biosystems, Foster City, CA, USA), according to the manufacturer’s procedure and TaqMan technology [40]. qRT-PCR specific for miR-143 was done using the TaqManH MiRNA Assays protocol (assay ID 002249; Applied Biosystems). Reverse transcriptase reaction was performed using 50 ng of total RNA and 50 nM miRNA specific stem-loop RT primers. All qRT-PCRs were run in triplicate as described previously [41]. Normalization was performed by using U6 snRNA primer kit (ID 001973, Applied Biosystems). Relative expression was calculated with relative standard curves for both the miRNA of interest and the endogenous control [41]. qRT-PCR analysis was performed using an ABI Prism 7900 Sequence Detector (Applied Biosystems).

### Western blot and antibodies

Since β-dystrobrevin may work as a scaffolding protein involved both in the activation and in the repression of neuron specific genes [20], we analyzed its expression at nuclear, cytoplasmic and total levels. NT2/D1 cells total protein extracts were prepared by using RIPA buffer (R0278, Sigma-Aldrich, St Louis, MO, USA) according to the manufacturer’s procedure. NT2/D1 cells cytoplasmic and nuclear extracts were prepared as previously described [42]. Briefly, the cells were lysed in a buffer containing 0.2% NP40, 10 mM HEPES pH 7.9, 10 mM KCl, 10 mM EDTA, 10mM EGTA, 1 mM DTT, and protease inhibitors, before centrifugation (1 min, 16.000 rpm) and supernatant collection (Cytoplasmic extract). The remaining pellets were lysed in 20 mM HEPES pH 7.9, 400 mM NaCl, 5 mM MgCl_2_, 1 mM EDTA, 1 mM EGTA, 25% Glycerol, 1 mM DTT, and protease inhibitors. After 15 min of incubation on ice, the nuclear extracts were centrifuged 15 min at 14,000 rpm and the supernatant was collected (Nuclear extract). Aliquots of 25–30 μg of protein extracts were resolved on SDS-PAGE and transferred to nitrocellulose filters. Blots were blocked for 1 h at room temperature in 5% nonfat-dry milk dissolved in TBS-T (10 mM Tris–HCl pH 8.0, 150 mM NaCl, 0.2% Tween 20) and then incubated with the primary antibody in 1% BSA/TBS-T overnight at 4°C. After several washes in TBS-T buffer, blots were incubated with the corresponding peroxidase-conjugated secondary antibody (Amersham Biosciences, Piscataway, NJ, USA) in TBS-T for 1 h. Bound antibodies were visualized by using the enhanced chemiluminescence technique (ECL) according to the manufacturer's instructions (Super Signal West Pico, Pierce, Rockford, IL, USA). Polyclonal antibodies (pAb) used were: anti-β-dystrobrevin [16], anti-HIF-1α (AF1935, R&D Systems, Minneapolis, MN, USA), anti-p27 (c-19) (sc-528, Santa Cruz Biotechnology, Bergheimer, Heidelberg, Germany), anti-synapsin I (106002, Synaptic Systems, Göttingen, Germany). A monoclonal anti-actin antibody (Sigma), for total and cytoplasmic extracts, and a monoclonal anti-nucleolin antibody (Oncogene Research Products, Boston, MA, USA), for nuclear extracts, were used as internal controls of the loaded amounts of proteins. The densitometry analysis was performed using a FluorChem E system, software version 4.1.1; the intensity of each band was normalized to that of the corresponding band of actin, or nucleolin.

### Luciferase target assay

We amplified by PCR the 3’UTR region of human β-dystrobrevin sequence (DTNB; NM_021907, from nucleotide 2148 to 2463), which includes the putative miR-143 target site, and sub-cloned the resulting 306 bp DNA product downstream of the Renilla luciferase gene into the psiCHECK-2 reporter vector (Promega, Madison, WI, USA), to create the reporter construct psiCHECK-2/3’UTR-β-DB (3’UTR-β-DB). By mutagenesis of the miR-143 binding site on β-dystrobrevin (from nucleotide 2407 to 2446, 5’-CATCACTCCCCTCAGGGCATGGTCTCATCTCCGCATCAGG-3’) in the reporter construct 3’UTR-β-DB, performed by using the QuickChange Site-Directed mutagenesis kit (Stratagene, La Jolla, CA, USA) and a primer with the mutated sequence of miR-143 target site (5’-CATCACTCCCCTCAGGGC***T***TG***AGGCTCGAGG***CGCATCAGG-3’), we created a mutated version of the 3’UTR-β-DB reporter construct (Mut-3’UTR-β-DB). Lipofectamine mediated co-transfections of 293T cells were performed by using 75 ng of the 3’UTR-β-DB or the Mut-3’UTR-β-DB construct and 60 pmol of either a non-targeting miRNA negative control (miRIDIAN Mimic Negative Control #1, Dharmacon, Lafayette, CO, USA) or miR-143 (miRIDIAN Mimic hsa-miR-143, Dharmacon), as described [41]. After 48 h, the cells were lysed with Passive Lysis Buffer (Promega) and luciferase activity was measured by using a Microlite TLX1 luminometer (Dynatech Laboratories, Chantilly, VA, USA). The relative reporter activity was obtained by normalization to the 3´UTR-β-DB construct/control oligonucleotide co-transfection.

### Overexpression of miR-143 in NT2/D1 cells

First, RA-untreated NT2/D1 cells were transfected by using lipofectamine, with siGLO Green Transfection Indicator and *(i)* miR-143 (miRIDIAN Mimic hsa-miR-143; 10 and 20 nM), or *(ii)* miR-C, a non-targeting miRNA negative control (miRIDIAN Mimic Negative Control #1; 10 and 20 nM). Two days after transfection, FITC-positive cells were isolated by cell sorting as previously described [41] and in part harvested for protein extraction and Western blot analysis of β-dystrobrevin and synapsin I protein expression; the remaining FITC-positive cells were maintained in culture and induced to proliferate and differentiate by RA treatment. miR-143, miR-C and siGLO Green Transfection Indicator were purchased from Dharmacon.

### miR-143 suppression in RA-NT2/D1 cells

Day 3 RA-treated NT2/D1 cells and day 5 RA-treated NT2/D1 cells were first transfected by using lipofectamine, as previously described [41], with anti-miR-143 (α-miR-143; miRIDIAN hsa-miR-143 hairpin inhibitor; 20 nM), or anti-miR-C (α-miR-C), a non-targeting anti-miRNA negative control (miRIDIAN microRNA Hairpin Inhibitor Negative Control). Then, transfected cells were cultured for 2 days in RA-containing medium before to be collected for analysis as: *(i)* total protein extracts from day 5 RA-treated NT2/D1(α-miR-143) cells and day 5 RA-treated NT2/D1(α-miR-C) cells; *(ii)* total protein extracts from day 7 RA-treated NT2/D1(α-miR-143) cells and day 7 RA-treated NT2/D1(α-miR-C) cells. α-miR-143 and α-miR-C were purchased from Dharmacon.

### Knockdown of β-dystrobrevin expression in NT2/D1 cells by RNA interference

The β-dystrobrevin gene was silenced with β-DB-synthetic small interfering ribonucleic acids (β-DB-siRNA; SMARTpool, ON-TARGETplus DTNB siRNA from Dharmacon). 1,5x10^6^ NT2/D1 cells were transfected with 50 nM of β-DB-siRNA, or an equal amount of non-targeting control siRNA (c-siRNA; ON-TARGETplus Non-targeting Control siRNA from Dharmacon) using lipofectamine according to the manufacturer's instruction. Two days after transfection, cells were in part harvested for mRNA and protein extraction to assess β-dystrobrevin and synapsin I expression by real time PCR and Western blot analysis; the remaining cells were maintained in culture and induced to proliferate and differentiate by RA treatment analysis. Day 2 RA-treated-transfected cells were also in part harvested for protein extraction and Western blot analysis of β-dystrobrevin protein expression.

### Statistical analysis

Unless otherwise indicated, results are presented as mean ± standard deviation of three independent experiments. Student t-test was used to calculate the statistical significance (P-value of more than 0.05 was considered statistically not significant).

## Results

### Hypoxia impairs RA-mediated neuronal differentiation of NT2/D1 cells

The human NT2/D1 cell line, which exhibits the properties of multipotent stem cells and differentiates into neurons on treatment with retinoic acid (RA) [37], is a well-established model for studying neurogenesis [43]. Since oxygen (O_2_) concentration has also been reported to be a crucial factor in growth and differentiation of neural cells [24], we have started our study by examining the effects of O_2_ concentration on the proliferation and differentiation of this cellular model.

**Fig 1:**
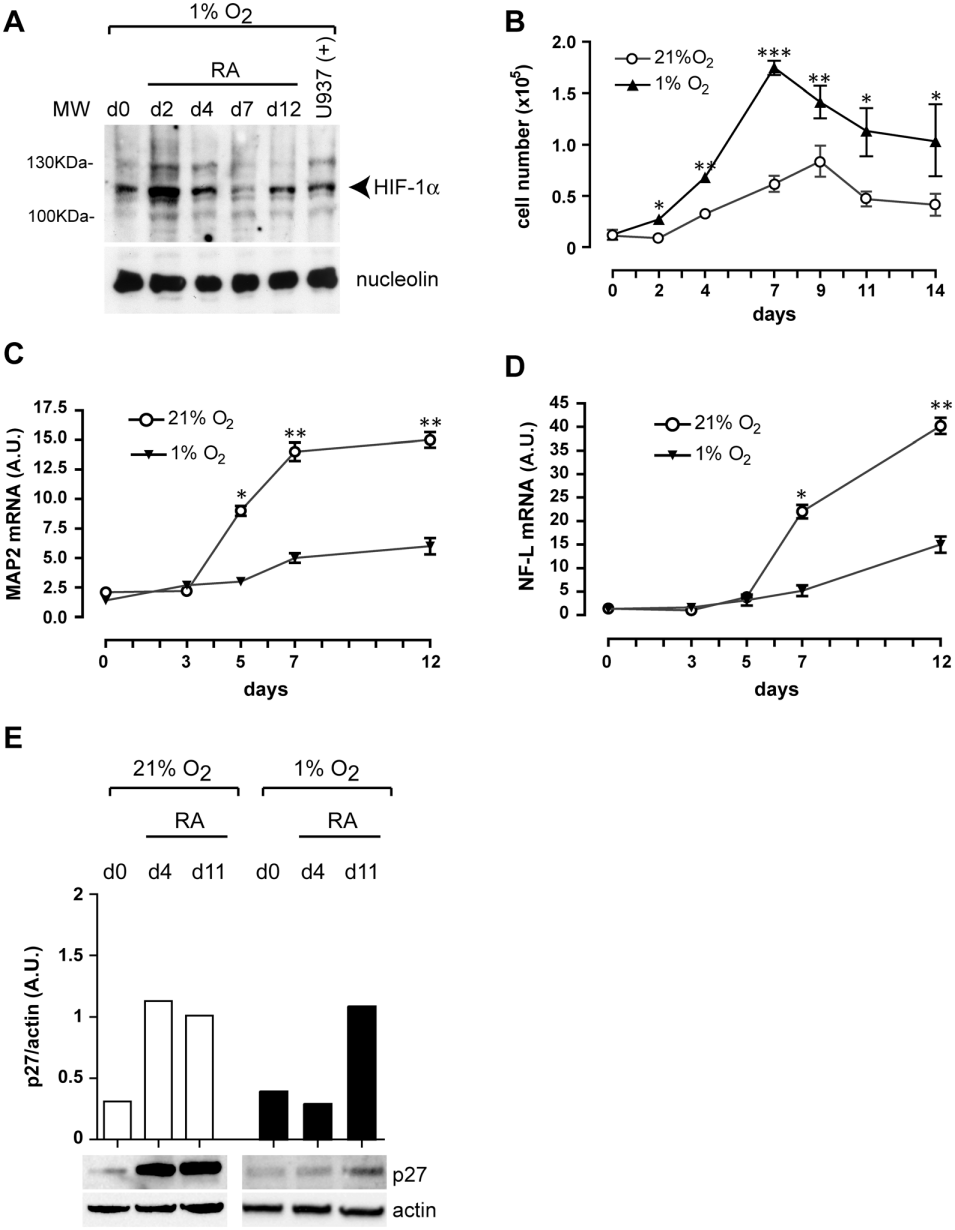
Hypoxia increases proliferation and impairs neuronal differentiation of RA-treated NT2/D1 cells. (**A**) Hypoxia (1% O_2_) activates HIF-1α nuclear protein expression in untreated (d0) and RA-treated NT2/D1 cells, as shown by Western blot analysis performed on nuclear extracts with a polyclonal HIF-1α antibody. (**B**) RA-treated NT2/D1 cells display a higher proliferation rate in hypoxia (1% O_2_) than in normoxia (21% O_2_), as shown by cell counting. (**C**, **D**) Real-time PCR analysis of mRNA expression of two neuron-specific genes, MAP2 (**C**) and NF-L (**D**), shows that RA-induced neuronal differentiation of NT2/D1 cells is impaired in hypoxia, compared with normoxia. (**E**) *Lower panels*: Western blot analysis of p27 impaired protein expression in RA-treated NT2/D1 cells in hypoxia, compared with normoxia. *Upper panel*: densitometry analysis of p27 protein expression levels compared with actin levels. (**B**, **C**, **D**) The results of three independent experiments (mean ± SEM values) are shown; *, **, *** represent p<0.05, p<0.01, p<0.001 respectively; the lack of error bars indicates that they are smaller than the symbol. (**A**, **E**) One representative experiment out of three is shown; (**A**) nucleolin is shown as internal control of nuclear protein extracts; U937(+) indicates nuclear extracts prepared from hypoxic U937 cells, used as positive control of HIF-1α nuclear protein expression; (**E**) actin is shown as internal control of total protein extracts. (**C**, **D**) A.U., arbitrary units.

We kept untreated NT2/D1 cells in culture for 2 days under normoxic (21% O_2_) or hypoxic (1% O_2_) conditions (day 0), and then, maintaining the different O_2_ concentrations, started the treatment with RA to induce neuronal differentiation. We verified the effect of hypoxia on these cells by following HIF-1α nuclear activation through Western blot on NT2/D1 nuclear extracts, and found that HIF-1α translocated into the nucleus in hypoxia (Fig 1A) but not in normoxia (not shown), as expected [44]. We, therefore, can exclude the possibility that RA might induce HIF-1α protein expression in normoxia.

While RA treatment of NT2/D1 cells in normoxia induces growth arrest and terminal differentiation along the neuronal pathway [37,45], we found that in hypoxia the cell proliferation rate of RA-treated NT2/D1 cells was higher than in normoxia (Fig 1B) and their neuronal differentiation impaired, as assessed by real-time PCR expression profiling of two neuron-specific genes, MAP2 (Fig 1C) and NF-L mRNAs (Fig 1D). Our observation was confirmed by Western blot analysis of p27 expression, a protein that is required for growth arrest and neuronal differentiation [46], whose expression appears to be impaired in hypoxic conditions (Fig 1E).

### β-dystrobrevin expression is regulated in RA-treated NT2/D1 cells

**Fig 2:**
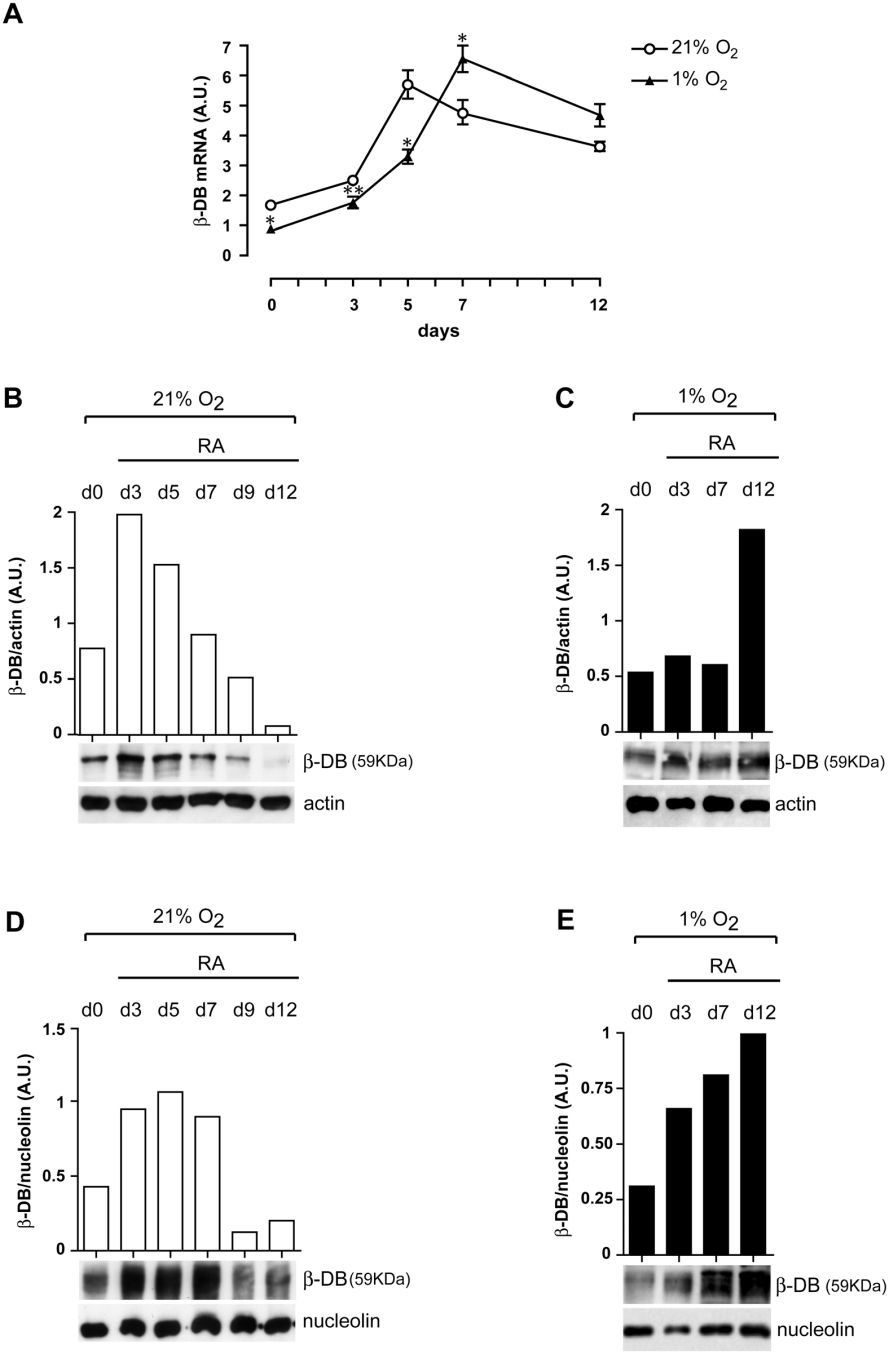
Hypoxia delays β-dystrobrevin expression during the early phase of RA-induced neuronal differentiation of NT2/D1 cells. (**A**) Real-time PCR analysis of β-dystrobrevin (β-DB) mRNA expression in untreated (d0) and RA-treated NT2/D1 cells under normoxic (21% O_2_) and hypoxic (1% O_2_) conditions. (**B**, **C**) *Lower panels*: Western blot analysis of β-DB total expression levels in untreated (d0) and RA-treated NT2/D1 cells, under normoxia (21% O_2_) (**B**) and hypoxia (1% O_2_) (**C**). *Upper panels*: densitometry analysis of β-DB total protein expression levels compared with actin levels. (**D**, **E**) *Lower panels*: Western blot analysis of β-DB nuclear protein expression levels in untreated (d0) and RA-treated NT2/D1 cells, under normoxia (21% O_2_) (**D**) and hypoxia (1% O_2_) (**E**); *Upper panels*: densitometry analysis of β-DB nuclear protein expression levels compared with nucleolin levels. (**A**) The results of three independent experiments (mean ± SEM values) are shown; *, ** represent p<0.05, p<0.01, respectively; the lack of error bars indicates that they are smaller than the symbol. A.U., arbitrary units. (**B, C, D, E**). One representative experiment out of three is shown. (**B**, **C**) actin is shown as internal control of total protein extracts; (**D**, **E**) nucleolin is shown as internal control of nuclear protein extracts.

We have investigated the expression of β-dystrobrevin in RA-treated NT2/D1 cells. In normoxia, β-dystrobrevin mRNA is expressed in untreated (day 0) NT2/D1 cells (Fig 2A, 21% O_2_), and RA stimulates its expression, which increases during the first days of treatment, from day 0 to 5, to decrease thereafter, as shown by real-time PCR analysis (Fig 2A, 21% O_2_). At the protein level, the expression of β-dystrobrevin, analyzed by Western blot on total cell extracts decreases after three days of RA treatment (Fig 2B). The expression pattern of the protein in respect to its mRNA suggests the possibility of post-transcriptional regulation.

In hypoxia, β-dystrobrevin mRNA expression pathway is delayed compared with normoxia, β-dystrobrevin mRNA levels peaking indeed at day 7 (Fig 2A, 1% O_2_), later than in normoxia. In the same interval of time, the protein is however consistently expressed, reaching its peak at day 12 (Fig 2C).

Having previously shown that β-dystrobrevin binds to and represses the promoter of the neuron-specific gene synapsin I in the nucleus of RA-treated NT2/D1 cells [20], we decided to analyze the expression pattern of β-dystrobrevin in nuclear extracts of untreated NT2/D1 cells and during RA-treatment of these cells. We found that in normoxia β-dystrobrevin nuclear expression rises during the first 5 days following RA treatment, and decreases thereafter (Fig 2D). At the same time points, in hypoxia, β-dystrobrevin nuclear expression is instead constantly upmodulated (Fig 2E).

We have also assessed β-dystrobrevin protein expression in the cytosol fraction. Compared with untreated NT2/D1 cells (day 0), β-dystrobrevin protein level increases in the cytosol fraction of day 5 RA-treated NT2/D1 cells, and decreases later after ten days of RA treatment, under normoxic conditions (S1A Fig). S1B Fig shows that cytosolic β-dystrobrevin protein expression is delayed in hypoxia, in line with the expression pattern of total or nuclear expression of β-dystrobrevin protein.

### β-dystrobrevin is a target gene of miR-143, a miRNA upregulated during RA-treatment of NT2/D1 cells

**Fig 3:**
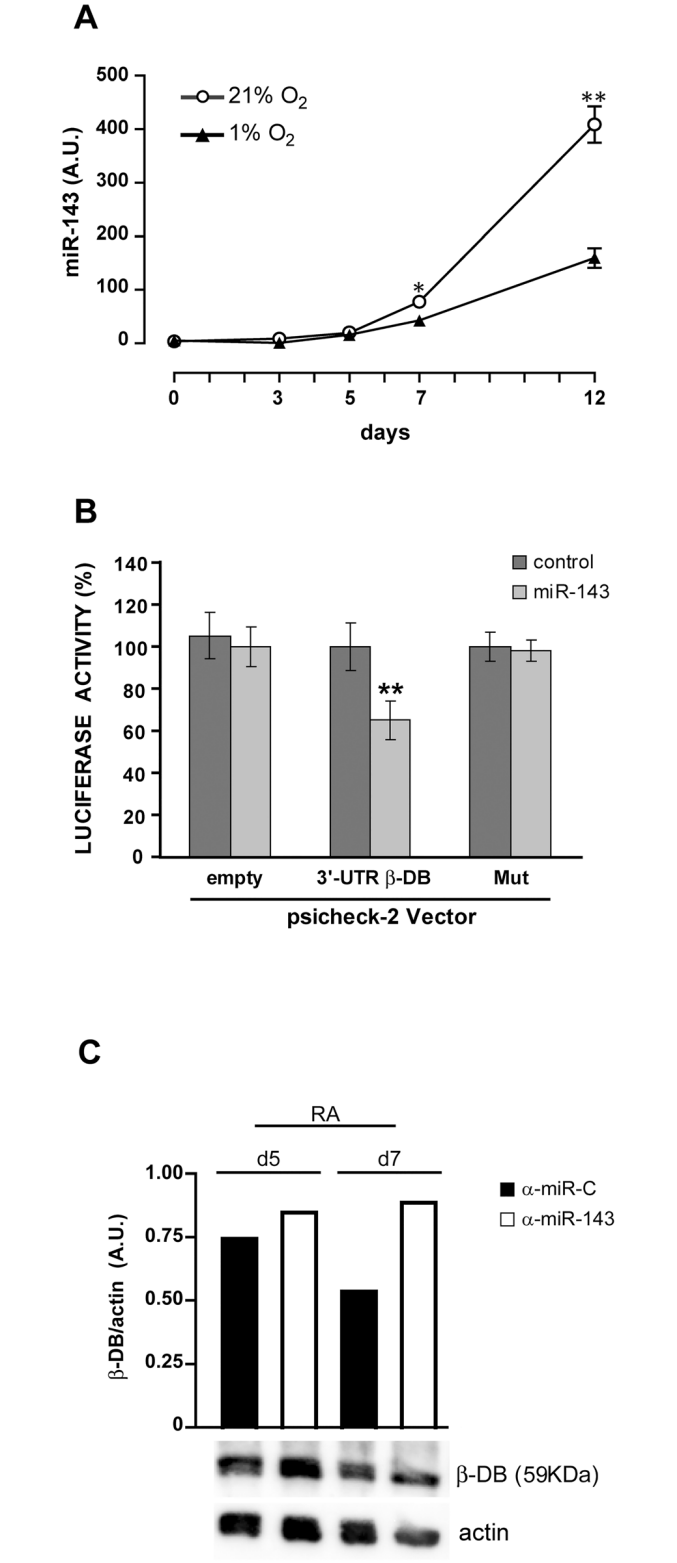
Hypoxia impairs expression of miR-143, a miRNA that directly targets β-dystrobrevin, in RA-treated NT2/D1 cells. (**A**) Real-time PCR analysis of miR-143 expression in untreated (d0) and RA-treated NT2/D1 cells under normoxic (21% O_2_) and hypoxic (1% O_2_) conditions. A.U., arbitrary units. (**B**) Luciferase targeting assays on 293T cells co-transfected with miR-143 or non-targeting miRNA negative control, and empty psicheck-2 vector or psicheck-2 vector carrying the full-length 3’UTR region of β-DB mRNA, wild type (3’-UTR-β-DB) or mutated to the miR-143 binding site (Mut). (**C**) *Lower panels*: Western blot analysis of β-DB protein expression levels in day 5 and day 7 RA-treated NT2/D1 cells transfected with anti-miR-143 (α-miR-143), compared with day 5 and day 7 RA-treated NT2/D1cells transfected with anti-miR of control (α-miR-C). *Upper panel*: densitometry analysis of β-DB protein expression levels compared with actin levels. (**A**, **B**) Data are the mean ± SEM values of three independent experiments. * and ** represent p<0.05 and p<0.01, respectively; the lack of error bars indicates that they are smaller than the symbol. (**C**) One representative experiment out of three is shown; actin is shown as internal control of total protein extracts.

In order to investigate whether the expression of β-dystrobrevin can be regulated at the translational level, we used bioinformatics tools to search for miRNAs that would target the β-dystrobrevin 3’-UTR. Through Target Scan 6.2 and miRanda predictions we found that β-dystrobrevin is a putative target gene of hsa-miR-143, a miRNA that has been reported to be involved in the mechanisms of cytoskeletal dynamics [47], response to hypoxia [48,49] and neuronal differentiation [50]. For this reason, we decided to analyze the expression of miR-143 during the RA treatment of NT2/D1 cells and found that, in line with a previous study conducted with different cell lines [51], miR-143 is upregulated in NT2/D1 cells following RA treatment (Fig 3A). In normoxia, miR-143 is indeed almost undetectable in untreated NT2/D1 cells (day 0) and during the first 5 days of RA treatment (Fig 3A, 21% O_2_), a period of time during which the cells are highly proliferating (Fig 1A, 21% O_2_). After day 5, the expression of miR-143 increases, particularly after day 7 (Fig 3A, 21% O_2_). These data inversely correlate with the expression pattern of β-dystrobrevin, which is downregulated at the same time points (Fig 2A, 21% O_2_ and 2B), suggesting that β-dystrobrevin could indeed be a target of miR-143. Interestingly, hypoxia impairs the upregulation of miR-143 (Fig 3A, 1% O_2_).

To verify whether β-dystrobrevin is a direct target of miR-143, we performed luciferase assays using miR-143 and reporter constructs carrying the wild-type β-dytrobrevin 3'UTR or the 3'UTR mutated at the putative miR-143 binding site. In 293T transfected cells, we found that miR-143 significantly repressed luciferase activity of the construct carrying the wild-type 3’UTR, compared with that carrying the mutated 3’UTR (Fig 3B), thus demonstrating that miR-143 binds to the 3'UTR of β-dystrobrevin mRNA.

### miR-143 controls β-dystrobrevin protein expression level and impairs proliferation of RA-treated NT2/D1 cells

To understand whether β-dystrobrevin protein expression level is regulated by miR-143 expression during RA-induced differentiation of NT2/D1 cells, we suppressed miR-143 expression and analyzed β-dystrobrevin protein expression in RA-treated NT2/D1 cells. We transiently transfected day 3 and day 5 RA-treated NT2/D1 cells with α-miR-143 or α-miR-C. 2 days after transfection, day 5 RA-treated NT2/D1(α-miR-143) and day 7 RA-treated NT2/D1(α-miR-143), with the respective α-miR-C-transfected control cells, were harvested and assessed for β-dystrobrevin levels by Western blot. We found that the suppression of miR-143 upregulates β-dystrobrevin protein expression in RA-treated NT2/D1(α-miR-143) cells compared with the respective control cells (Fig 3C). Our results demonstrate that β-dystrobrevin is a direct target of miR-143 in live cells and confirm that β-dystrobrevin expression is regulated by miR-143 during RA-induced neural differentiation of NT2/D1 cells.

**Fig 4:**
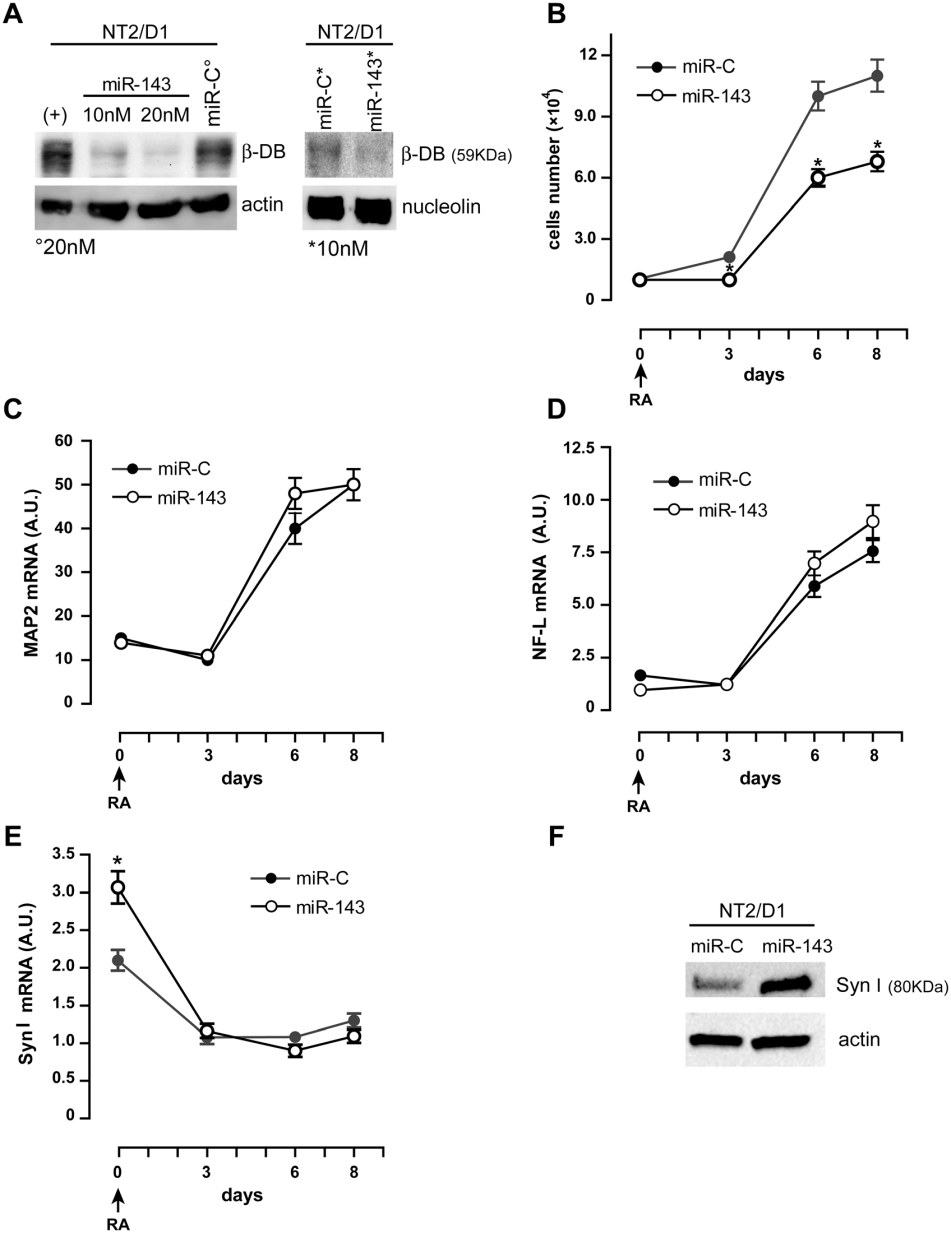
miR-143 overexpression affects proliferation of RA-treated NT2/D1 cells, decreases β-dystrobrevin protein level and increases synapsin I gene expression. (**A**) Western blot analysis of β-DB total (*left panels*) and nuclear (*right panels*) protein expression level in NT2/D1 cells transiently transfected with miR-143 (miR-143), as compared with untransfected NT2/D1 control cells (+) and NT2/D1 cells transiently transfected with a non-targeting negative control miRNA (miR-C); the degree symbol (°) indicates a concentration of 20 nM, the asterisk (*) represents 10 nM. (**B**) Cell proliferation analysis of RA-treated miR-143 transiently transfected-NT2/D1 cells compared with miR-C control cells. (**C**, **D**) Real-time PCR analysis of MAP2 (**C**) and NF-L (**D**) mRNA expression in RA-treated miR-143 transiently transfected-NT2/D1 cells compared with control cells. (**E**) Real-time PCR analysis of synapsin I (SynI) mRNA expression in RA-treated miR-143 transiently transfected-NT2/D1 cells compared with control cells. (**F**) Western blot analysis of SynI protein expression level in miR-143 transiently transfected-NT2/D1 cells (miR-143), compared with NT2/D1 control cells (miR-C). (**A**, **F**) One representative experiment out of three is shown; actin and nucleolin are shown as internal control of total and nuclear protein extracts, respectively; transfections were performed with 10 nM miRNAs, unless otherwise specified. (**B**-**E**) The results of three independent experiments (mean ± SEM values) are shown; * represents p<0.05; the lack of error bars indicates that they are smaller than the symbol. Arrow indicates the start of treatment with RA of the transfected NT2/D1 cells (day 0); (**C**-**E**) A.U., arbitrary units.

To shed further light on the functional role of miR-143, we transiently cotransfected NT2/D1 cells with a FITC-conjugated non-targeting oligonucleotide plus either miR-143 (NT2/D1-miR-143) or a non-targeting miR-C used as negative control (NT2/D1-miR-C). We used fluorescence-activated cell sorting (FACS) to select and collect FITC-positive cells and assessed the transfection efficiency to be around 70% (not shown). We harvested an aliquot of transfected cells to assess the expression of β-dystrobrevin by Western blot analysis, and induced to differentiate the remaining transfected NT2/D1-miR-143 and NT2/D1-miR-C cells by RA treatment. The overexpression of miR-143 in untreated NT2/D1 cells downregulates β-dystrobrevin protein at both total (Fig 4A, left panel) and nuclear (Fig 4A, right panel) level, compared with untreated NT2/D1-miR-C and untransfected NT2/D1 (+) control cells, thus confirming that β-dystrobrevin expression is affected by miR-143 targeting in live cells (Fig 4A). After RA treatment, we found that the cell proliferation rate of NT2/D1-miR-143 cells was significantly decreased compared with NT2/D1-miR-C control cells (Fig 4B), while the differentiation program does not seem to be affected, as we did not detect any significant variation in the mRNA expression of MAP2 (Fig 4C), or NF-L (Fig 4D).

Interestingly, we found that the expression of synapsin I was upregulated at both the mRNA and protein levels in untreated (day 0) NT2/D1-miR-143 cells (Fig 4E and 4F), when miR-143 is transiently overexpressed and β-dystrobrevin expression downregulated (Fig 4A).

### Synapsin I is a new target gene of β-dystrobrevin, regulated in RA-treated NT2/D1 cells

In a previous work, we demonstrated a direct interaction of β-dystrobrevin with the HMG20 proteins iBRAF and BRAF35, two chromatin remodeling proteins involved in the control of the expression of a number of neural genes. In the same paper, we also showed in vitro that β-dystrobrevin binds to and represses the promoter of synapsin I [20]. Here, having shown that of β-dystrobrevin nuclear protein expression is regulated during RA-induced differentiation of NT2/D1 cells (Fig 2D and 2E), we decided to analyze the expression of synapsin I mRNA in the same cells in order to confirm the transcriptional repressor role of β-dystrobrevin on synapsin I expression.

**Fig 5:**
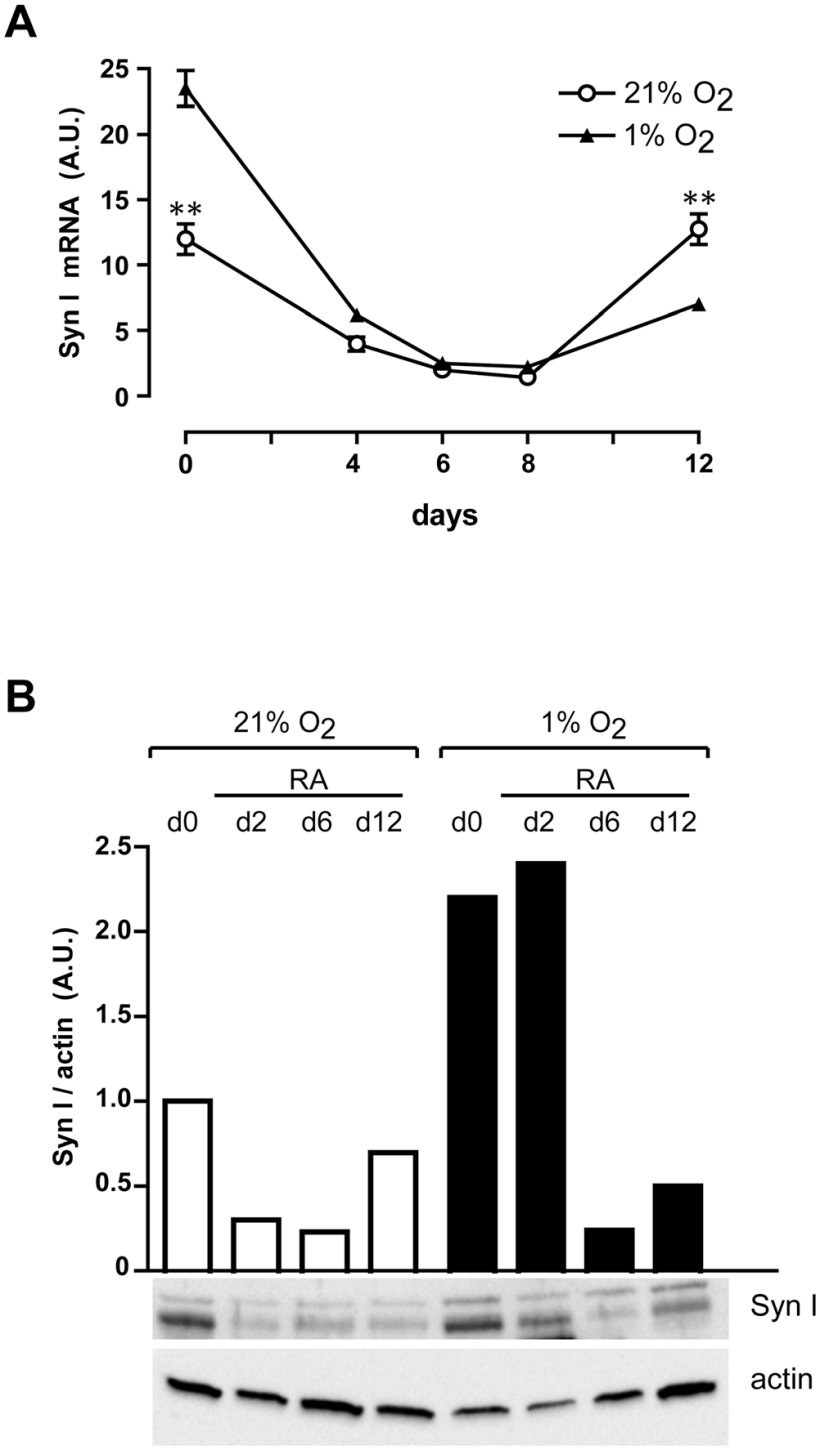
Hypoxia affects synapsin I expression in NT2/D1 cells. (**A**) Real-time PCR analysis of SynI mRNA expression in untreated (d0) and RA-treated NT2/D1 cells under normoxic (21% O_2_) and hypoxic (1% O_2_) conditions. (**B**) *Lower panels*: Western blot analysis of SynI protein expression levels in untreated (d0) and RA-treated NT2/D1 cells, under normoxic (21% O_2_) and hypoxic (1% O_2_) conditions. *Upper panel*: densitometry analysis of SynI protein expression levels compared with actin levels. (**A**) The results of three independent experiments (mean ± SEM values) are shown; ** represents p<0.01; the lack of error bars indicates that they are smaller than the symbol. A.U., arbitrary units. (**B**) One representative experiment out of three is shown; actin is shown as internal control of total protein extracts.

In normoxia, synapsin I mRNA, which is already expressed in untreated (day 0) NT2/D1 cells, decreases during the first days of RA treatment, remaining at quite a low level until day 8, and rises thereafter (Fig 5A, 21% O_2_). The expression of synapsin I protein follows that of its mRNA (Fig 5B, 21% O_2_). In hypoxia, synapsin I mRNA and protein levels follow the same pattern but are higher than in normoxia in NT2/D1 untreated cells (day 0) and during the first days of RA treatment (Fig 5A and 5B, 1% O_2_). After day 8 the expression of synapsin I rises but does not reach the level of expression shown in normoxia. The opposite expression pattern displayed by β-dystrobrevin nuclear protein expression (Fig 2D and 2E) and synapsin I mRNA expression (Fig 5A) during RA treatment of NT2/D1 cells, in normoxia as well as hypoxia, implies a transcriptional control of β-dystrobrevin on synapsin I gene expression. These data are also in line with our results in miR-143-transfected NT2/D1 cells in normoxia, where the decrease of β-dystrobrevin protein level (Fig 4A) well correlates with the increase of synapsin I expression (Fig 4E and 4F) during the short-term overexpression of miR-143.

### β-Dystrobrevin is directly involved in cell proliferation during the early phase of neural differentiation of NT2/D1 cells and regulates synapsin I gene expression

**Fig 6:**
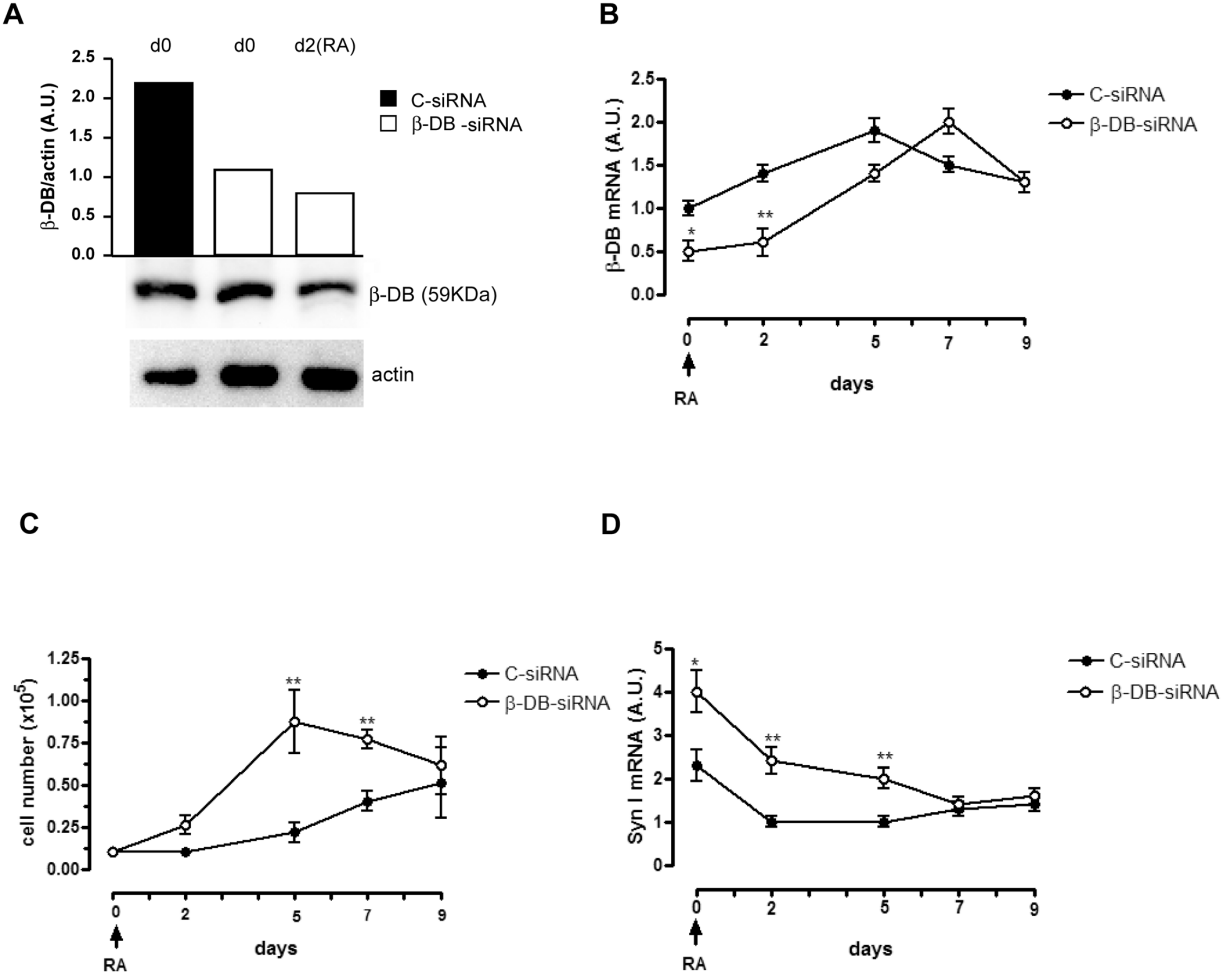
β-Dystrobrevin gene silencing increases cell proliferation and downregulates synapsin I expression during the early stage of RA-induced neuronal differentiation of NT2/D1 cells. (**A**) *Lower panels*: Western blot analysis of β-DB protein expression levels in untreated (d0) and day 2 RA-treated NT2/D1 cells transfected with β-DB-siRNA, compared with untreated (d0) NT2/D1 cells transfected with non-targeting control siRNA (C-siRNA). *Upper panel*: densitometry analysis of β-DB protein expression levels compared with actin levels. (**B**) Real-time PCR analysis of β-DB mRNA expression during RA treatment of β-DB-siRNA transfected-NT2/D1 cells compared with C-siRNA transfected cells. (**C**) Cell proliferation analysis of β-DB-siRNA transfected-NT2/D1 cell during RA treatment compared with C-siRNA transfected NT2/D1 cells treated with RA. (**D**) Real-time PCR analysis of Syn I mRNA expression during RA treatment of β-DB-siRNA transfected-NT2/D1 cells compared with C-siRNA transfected cells. (**A**) One representative experiment out of three is shown; actin is shown as internal control of total protein extracts. (**B**, **C**, **D**) Arrow indicates the start of treatment with RA of the transfected NT2/D1 cells (day 0); the results of three independent experiments (mean ± SEM values) are shown; *, ** represent p<0.05, p<0.01; the lack of error bars indicates that they are smaller than the symbol. A.U., arbitrary units.

To analyze further the role that β-dystrobrevin plays during the early stages of RA-treatment of NT2/D1 cells, we performed siRNA-mediated β-dystrobrevin knockdown experiments in our cellular system. We found that the early decrease of β-dystrobrevin mRNA and protein expression, assessed in NT2/D1(β-DB-siRNA) cells not treated with RA (day 0) and NT2/D1(β-DB-siRNA) RA-treated cells (day 2) (Fig 6A and 6B), induce an increase of cell proliferation (Fig 6C) and synapsin I mRNA expression (Fig 6D) during RA- differentiation of these cells; we did not detect any modulation of NF-L and MAP2 expression (not shown). These results demonstrate that β-dystrobrevin is directly involved in cell proliferation during the early phase of neural differentiation of NT2/D1 cells, and that synapsin I is a direct target gene of beta-DB at this stage.

## Discussion

Aiming at shedding light on the neuronal involvement in Duchenne muscular dystrophy (DMD), we have here investigated the possibility of a functional role of β-dystrobrevin in the molecular mechanisms underlying the first steps of neural differentiation. DMD, a genetic disorder that results in a gradual breakdown of muscle fibers, is often associated with cognitive deficits that are independent from the muscular pathology. The brain dysfunction occurs early, probably during embryogenesis, and most likely involves defects in the assembly of the DPC, whose components are emerging as major players in brain development and disease [6]. In a previous paper, we demonstrated the interactions of β-dystrobrevin with two chromatin remodeling proteins, iBRAF and BRAF35 [21,22], whose interplay is involved in the regulation of neuronal gene expression [52]. We also described the binding of β-dystrobrevin to the promoter of synapsin I [20], a neuron-specific protein whose alterations are known to be involved in a number of CNS disorders [36]. These observations prompted us to suggest that β-dystrobrevin may play a role in the events that lead to initiation of neuronal differentiation [20]. We have therefore analyzed the expression, regulation and function of β-dystrobrevin during the early stages of RA-mediated differentiation of NT2/D1 cells, a well-established cellular model of neural development [38]. We observed that the β-dystrobrevin mRNA expression increases during the initial phase of proliferation and decreases when the RA-induced differentiation of NT2/D1 cells is taking place. We also found that β-dystrobrevin protein levels do not seem to be strictly correlated with its mRNA levels, suggesting a post-transcriptional control of the β-dystrobrevin gene.

In order to elucidate this hypothesis, we used algorithmic analysis to test whether post-transcriptional regulation of β-dystrobrevin expression might be mediated by miRNA targeting, and identified miR-143 as a strong candidate for this role. That β-dystrobrevin mRNA translation is affected by miR-143 targeting has indeed been confirmed by the results of luciferase assays showing a direct binding of miR-143 to the β-dystrobrevin 3'UTR, and by a reduction, or respectively an increase of β-dystrobrevin protein expression level in miR-143- or anti-miR-143-transfected NT2/D1 cells. Altogether, these data demonstrate that β-dystrobrevin is a novel target gene of miR-143 during the early stages of neural differentiation of NT2/D1 cells.

In an effort to analyze the differentiation pattern of RA-treated NT2/D1 cells, we focused on the expression of some neuron-specific genes, among which synapsin I. Although fully differentiated RA-NT2/D1 cells show a significant transcriptional upregulation of synapsin [53], a study based on immunoblot assays claimed that a short-term RA-treatment was not sufficient to induce the expression of synapsin I or the other synaptic vesicle-associated proteins [54]. Here we demonstrated that synapsin I expression is already detectable in untreated NT2/D1 cells, but it rapidly decreases during the first days of RA treatment, at the same time points where β-dystrobrevin nuclear expression rises. It is of interest to note that here, for the first time, we describe the nuclear localization of β-dystrobrevin protein in NT2/D1 cells, confirming what was previously reported in other cell lines [55–57] or primary cell models [58]. Our β-dystrobrevin silencing experiments confirmed that β-dystrobrevin plays a role as repressor of synapsin I gene expression and is involved in cell proliferation during the very early stage of neural differentiation of NT2/D1 cells, when endogenous miR-143 expression is low. Indeed, in a previous paper we reported that β-dystrobrevin binds to and represses the promoter of synapsin I, suggesting that dystrobrevin might play a role in differentiation processes as a component of co-activator/co-repressor complexes required for the regulation of neural-specific genes [20]. Likewise, in neuroblastoma Neuro2a cells, it was recently demonstrated that the transcriptional activator Sp1 directly binds the synapsin I promoter, activating its transcription, and that its activity is directly modulated by the neural master regulator REST [59].

In the context of neural differentiation, oxygen concentration has been reported as a crucial factor [24], implying that understanding how manipulate hypoxia may lead to novel strategies for therapeutic intervention [23,24]. Cellular adaptation to hypoxia involves numerous mechanisms, including the transcriptional activation mediated by HIF-1α of genes and miRNAs, which in concert with their target genes and classical transcriptional factors regulate neuronal development, function and plasticity [60–62]. Here we report that in hypoxia the cell proliferation rate of RA-treated NT2/D1 cells increases while neuronal differentiation is impaired, as compared with normoxia. Similar data have been recently described also for neural stem cells, prompting the hypothesis that low O_2_ in the body may give rise to stem cell niches, where spontaneous differentiation is suppressed and thus a great stem cell pool engendered [63]. In hypoxia, during RA-induced differentiation of NT2/D1 cells, β-dystrobrevin expression pathway and miR-143 upregulation are delayed, as compared with normoxia. In the same condition, while β-dystrobrevin protein levels are lower, synapsin I mRNA and protein levels are higher compared with the levels in normoxia, thus providing additional evidence for a regulatory pathway that involves mir143, β-dystrobrevin and synapsin I.

Our results, obtained using a reproducible experimental model of neurogenesis, suggest that the regulated expression of β-dystrobrevin mediated by miR-143 might be relevant for a correct assembly and function of DPC-like-complexes in brain, and possibly involved in the establishment of pathological conditions like the cognitive impairment associated to muscular dystrophy. To the best of our knowledge, ours is the first study to report a miRNA-based regulation of DPC components during neural differentiation of human cells. Recent papers have identified several circuitries implicating DPC components and miRNAs that are relevant for the DMD pathogenesis [64–66]; similar mechanisms have also been suggested to be present in neuronal tissues [32]. Recently, miRNAs have emerged as important modulators in neural lineage determination [67,68], and in this context our results may contribute in shedding light on strategies to optimize neuronal differentiation in vitro for use in drug discovery and novel therapeutic applications.

Altogether, our data provide new insights into the role of β-dystrobrevin in the molecular mechanisms underlying neuronal differentiation that could be relevant in the establishment of the cognitive impairment in DMD. We need to investigate further whether miR-143 may be a valid tool to manipulate β-dystrobrevin expression during proliferation and neuronal differentiation. Since miRNAs have been implicated in neurodevelopmental and neuropsychiatric disorders [30,31] and alterations of synapsin I expression are known to be involved in a number of CNS disorders [34,35], a better understanding of the newly identified regulatory pathway miR-143/β-dystrobrevin/synapsin I in NT2/D1 cells may be useful in providing information that can help define specific targets for pharmacological approaches.

## Supporting Information

S1 Fig β-Dystrobrevin cytosolic protein expression is regulated during RA-induced differentiation of NT2/D1 cells in normoxic and hypoxic conditions. (**A**, **B**) *Lower panels*: Western blot analysis of β-DB cytosolic protein expression in untreated (d0) and RA-treated NT2/D1 cells, under normoxia (21% O_2;_ **A**) and hypoxia (1% O_2;_ **B**); *Upper panels*: densitometry analysis of β-DB cytosolic protein expression levels compared with actin levels. (**A, B**) One representative experiment out of three is shown; actin is shown as internal control of cytosolic protein extracts; A.U., arbitrary units.
(TIF)
