# Supplementary figures and images for: Identification of β-Dystrobrevin as a Direct Target of miR-143: Involvement in Early Stages of Neural Differentiation
Source: PLoS One. 2016 May 25;11(5):e0156325. doi: 10.1371/journal.pone.0156325 (PMC4880309; doi:10.1371/journal.pone.0156325)

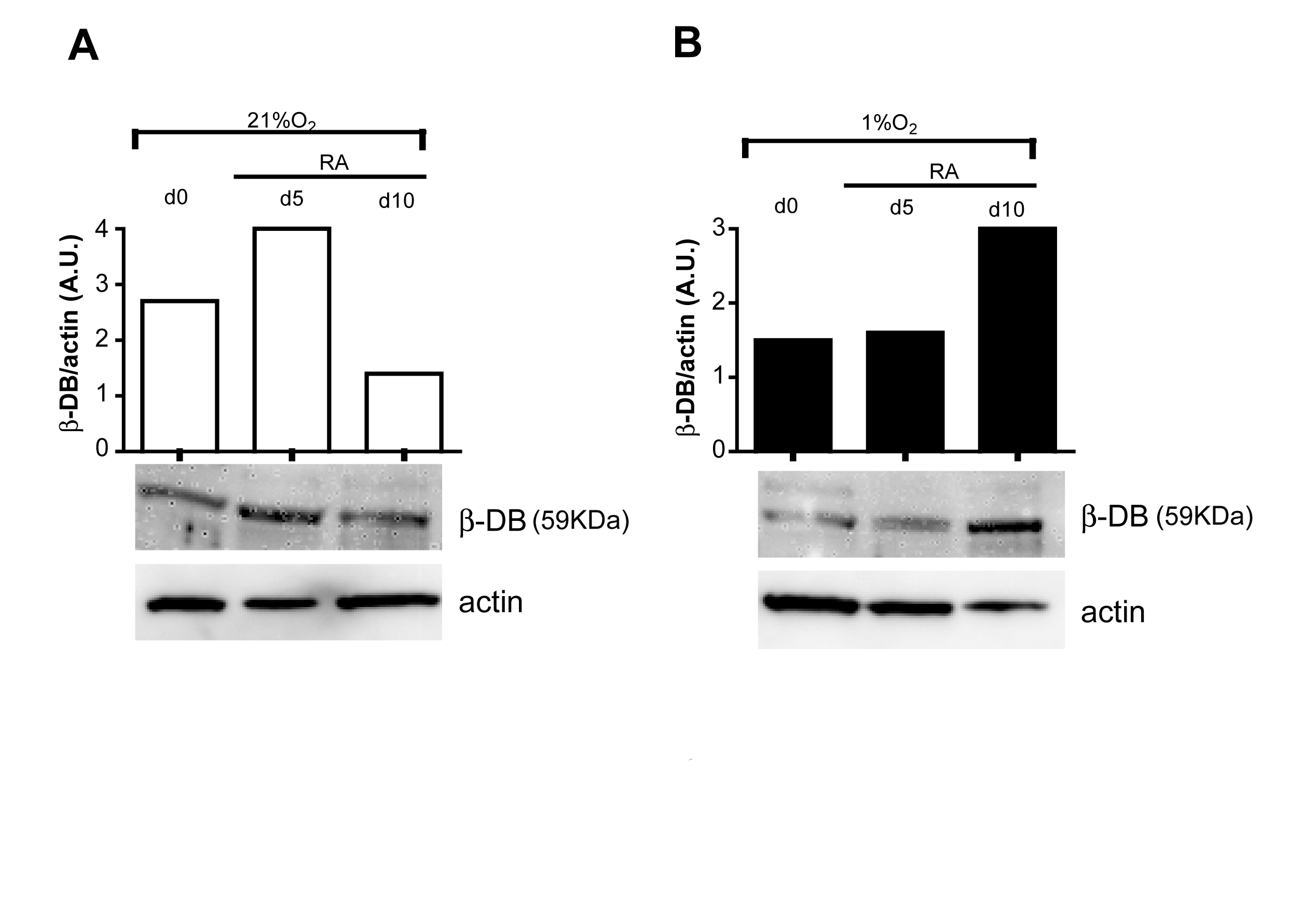

Supplement: S1 Fig — (A, B) Lower panels: Western blot analysis of β-DB cytosolic protein expression in untreated (d0) and RA-treated NT2/D1 cells, under normoxia (21% O2; A) and hypoxia (1% O2; B); Upper panels: densitometry analysis of β-DB cytosolic protein expression levels compared with actin levels. (A, B) One representative experiment out of three is shown; actin is shown as internal control of cytosolic protein extracts; A.U., arbitrary units. (TIF) [file pone.0156325.s001.tif]
